# Accelerated aging syndromes, are they relevant to normal human aging?

**DOI:** 10.18632/aging.100383

**Published:** 2011-09-14

**Authors:** Oliver Dreesen, Colin L. Stewart

**Affiliations:** Institute of Medical Biology, 8A Biomedical Grove, #06-06 Immunos, 138648 Singapore

**Keywords:** Aging, Hutchinson-Gilford Progeria, Werner syndrome, DNA damage, telomeres, Wnt signaling, Lef1

## Abstract

Hutchinson-Gilford Progeria (HGPS) and Werner syndromes are diseases that clinically resemble some aspects of accelerated aging. HGPS is caused by mutations in theLMNA gene resulting in post-translational processing defects that trigger Progeria in children. Werner syndrome, arising from mutations in the WRN helicase gene, causes premature aging in young adults. What are the molecular mechanism(s) underlying these disorders and what aspects of the diseases resemble physiological human aging? Much of what we know stems from the study of patient derived fibroblasts with both mutations resulting in increased DNA damage, primarily at telomeres. However, in vivo patients with Werner's develop arteriosclerosis, among other pathologies. In HGPS patients, including iPS derived cells from HGPS patients, as well as some mouse models for Progeria, vascular smooth muscle (VSM) appears to be among the most severely affected tissues. Defective Lamin processing, associated with DNA damage, is present in VSM from old individuals, indicating processing defects may be a factor in normal aging. Whether persistent DNA damage, particularly at telomeres, is the root cause for these pathologies remains to be established, since not all progeroid Lmna mutations result in DNA damage and genome instability.

## INTRODUCTION

Age is the major risk factor in the development of many chronic medical conditions including cancer [1,2]. To enhance the well-being of an increasingly aged population, and to identify new avenues for therapeutic intervention, it is crucial that we understand the molecular basis underlying aging and age-related diseases. Aging can be defined as the “progressive deterioration of virtually every bodily function over time” [3] ultimately resulting in death. At the cellular level, aging is associated with an increase in DNA lesions, together, with defects in DNA repair mechanisms. Of particular relevance is DNA damage associated with critically shortened telomeres. Telomeres, which cap the ends of chromosomes, consist of hexameric TTAGGG repeats and the protective “shelterin” protein complex [4]. Due to the “end replication problem”, telomeres shorten during each replication cycle and critically shortened telomeres elicit a persistent DNA damage response that triggers an irreversible growth arrest (senescence). Shortened telomeres limit the regenerative capacity of tissues and are associated with increased age and a variety of medical conditions including Dykeratosis Congenita, aplastic anemia, and pulmonary fibrosis [5,6]. In germ cells, stem cells and ~85% of cancers, telomere length is maintained by telomerase, a ribonucleoprotein consisting of a reverse transcriptase (*TERT*) and its RNA moiety (*TERC*); expression of telomerase renders cells immortal.

Two segmental premature aging diseases that clinically appear to present as accelerated aging in some tissues, are Werner syndrome and Hutchinson-Gilford Progeria (HGPS). In both diseases recent evidence indicates that mutations in the genes responsible for these premature aging diseases result in increased DNA damage, particularly at telomeres. Although shortening and/or damage to telomeres is associated with proliferative arrest of cells *in vitro*, it remains unclear how accurately these diseases recapitulate the processes of tissue aging in humans. Here we discuss recent advances, using *in vitro* cell culture and mouse models of progeroid syndromes to highlight important questions that remain: A) what is the molecular mechanism of how such seemingly unrelated proteins cause similar degenerative diseases? B) are these mechanisms representative of normal aging?

### Werner Syndrome and Hutchinson-Gilford Progeria

Werner syndrome is caused by mutations in the Werner RecQ helicase, a DNA helicase/exonuclease [7] whereas HGPS is caused by mutations in Lamin A (*LMNA*) [8,9]. The Wrn RecQ helicase is involved in repair of double strand DNA breaks and in faithful replication of lagging-strand telomeres. In its mutated form, *WRN* causes sporadic loss of telomeres generated by lagging strand synthesis, increased DNA damage (presumably caused by critically shortened telomere(s)), premature senescence and genomic instability [10,11]. These phenotypes are suppressed in the presence of telomerase [10,12,13]. In Werner syndrome, telomere dysfunction is causal to the accumulation of DNA damage foci and results in premature senescence *in vitro.* These defects may, therefore, underlie the *in vivo* disease etiology.

The A-type lamins are nuclear intermediate filament proteins that form the nuclear lamina. The lamina underlies the inner nuclear membrane (INM) and is important in maintaining interphase nuclear shape, mechanical integrity and also functions as a scaffold for other nuclear proteins, some of which regulate DNA replication and transcription. The most intriguing aspect of the lamins, particularly LaminA, is that some dozen or so diseases, the laminopathies, are caused by different mutations in the *Lmna* gene. These diseases affect tissues primarily of mesenchymal origin, resulting in defective muscle and cardiac function, fat deposition, skeletal homeostasis and vascular integrity [14].

A critical feature of LaminA is that the protein undergoes significant post-translational processing. The processing involves farnesylation of the C-terminal cysteine, which is thought to promote the association of the LaminA protein with the INM. The farnesylated LaminA protein then undergoes two proteolytic cleavage steps, by the endoprotease ZMPSTE24, culminating in the removal of the farnesyl group and the 18 C-terminal amino acids. In HGPS, the most common cause is a point mutation in the *LMNA* gene, which creates a novel splice site, removing 50 amino acids from the C-terminal globular domain. The deletion includes the ZMPSTE24 endoproteolytic site, resulting in the synthesis of a truncated, farnesylated form of LaminA, called Progerin [8,9]. To what extent the deletion and/or the persistent farnesylation is the molecular basis to HGPS is unclear, as a mouse model of HGPS expressing the truncated, but non-farnesylated variant of Progerin still develop progeroid symptoms [15] and non farnesylated Progerin in human cells elicits the same DNA damage responses as farnesylated Progerin [16]. In addition, other patients, diagnosed with Progeria or, intriguingly with atypical Werner's syndrome, have mis-sense mutations elsewhere in the *Lmna* gene and it seems unlikely that these mutations affect endoproteolytic cleavage or farnesyl processing, although this needs to be confirmed [17]. Progerin results in abnormal nuclear morphologies, altered chromatin organization, delayed mitosis, lamina thickening, and growth arrest [18-20]. However increasing evidence suggests that at least one underlying cause is that Progerin damages telomeres.

First, and in parallel with their limited proliferative capacity, progeric fibroblasts have significantly shorter telomeres than age-matched controls [21,22]. Secondly, expressing Progerin in wild-type fibroblasts gradually inhibits their proliferation. Strikingly, proliferative inhibition is reversed by telomerase activation and to some extent by p53-deficiency [23], suggesting that Progerin directly or indirectly damages telomeres, thus activating p53, but the damage is reversed by telomerase. Thirdly, persistent activation of DNA damage checkpoints and increased numbers of DNA damage foci are present in HGPS-patient derived fibroblasts [16,24,25]. Increased DNA damage is a classic feature of cells undergoing senescence due to shortened or deprotected telomeres [26,27]. The DNA damage foci co-localize with human TRF1, a component of the shelterin complex, and the DNA damage sensor H2A-X is also expressed and enriched at telomeric DNA [28]. In addition, live cell imaging and cytometric analysis indicate that telomeres in Progeria patient-derived cells are hypermobile [29], a characteristic of de-protected telomeres [30]. Lastly, many of these Progerin induced defects, are suppressed by expression of telomerase in fibroblasts [23,28].

Together, increased DNA damage, as well as the shortened telomeres in HGPS, are considered as characteristics of normal human aging. However a significant difference is that during normal aging, telomere dysfunction is mainly a consequence of the end replication problem, which may also be enhanced by DNA damaging agents such as oxidative stress [31]. In HGPS, telomeres may be directly damaged by Progerin. However, the precise mechanism by which telomeres are damaged, and whether only a subset of telomeres, such as those located at the nuclear periphery, still needs to be established. How well do these *in vitro* cell culture results recapitulate the *in vivo* pathologies in patients and mouse models of Progeria?

### Mouse models of HGPS and Werner syndrome

The role of DNA damage resulting in accelerated aging has been recapitulated in a number of mouse models (reviewed by Schumacher *et al*. [32]). One example is the XPF-ERCC1-deficient mouse. XPF-ERCC1 is required for nucleotide excision repair, and repairs DNA lesions due to UV radiation. XPF-ERCC1-deficient mice exhibit slightly retarded embryonic and post-natal development, followed by growth arrest by ~2 weeks, and death at ~4 weeks after birth. Phenotypically, these mice show aged-like phenotypes in skin, liver and bone marrow. XPF-ERCC1 cells prematurely senesce and are more sensitive to oxidative stress [33]. Two mouse models for Werner syndrome were generated by deletion of the *Wrn* gene, but neither recapitulated any accelerated aging phenotypes observed in humans or in cultured cells [34,35]. This discrepancy was thought to be due to laboratory mice having extremely long telomeres and residual levels of telomerase in some somatic tissues [36,37]. Accordingly, the additional deletion of *terc* (the RNA component of telomerase) in *Wrn* mice resulted in shortened telomeres, with the appearance of the classic phenotypes of accelerated aging, i.e. grey hair, osteoporosis, alopecia and premature death [38]. Embryonic fibroblasts (MEFs) from these mice exhibited genomic instability, increased levels of DNA damage and senesced prematurely, corroborating previous results [38]. Interestingly, in contrast to telomerase-deficient mice, in which the degenerative phenotypes manifest mainly in highly proliferative tissues (intestine, skin and blood), in *Wrn*^−/−^/*Terc*^−/−^ mice, tissues of mesenchymal origin were mainly affected resulting in osteoporosis, cataracts and diabetes [38].

Several HGPS-progeroid mouse models have been established to investigate how *Lmna* mutations, including defective processing of normal Lamin A, may accelerate aging [39-45]. The first *Lmna* mutant showing premature aging was generated by a splicing defect deleting exon 9, resulting in an in frame deletion of 40 amino acids in the C-terminal domain (*Lmna*^L530P/L530P^; also called ∆9*Lmna*) [39]. The mutation, like Progerin, results in a truncated variant of LaminA that remains farnesylated, although the ∆9 protein is expressed at about 10% that of wild-type levels. At birth, homozygotes were indistinguishable from their wild-type littermates. However, post-nataly, they exhibit a rapid onset of severe growth retardation, loss of subcutaneous fat, poor heart development, skeletal abnormalities, decreased hair follicle density, culminating in death at 3-4 weeks. Postnatal ∆9*Lmna* fibroblasts (MAFs), established from several different tissues, show a highly restricted proliferative capability with early death. Although ∆9*Lmna* MAFs do exhibit abnormal nuclear morphologies, premature death of the cells was not associated with overt chromosomal defects such as aneuploidy or increased DNA damage. Loss of the MAFs proliferative capability was due to reduced synthesis of some 30 extracellular matrix proteins (ECM), since serial culture of the ∆9*Lmna* fibroblasts on ECM deposited by normal MAFs sustained their proliferation. Surprisingly, and in contrast to the MAFs, the growth properties of ∆9*Lmna* embryonic fibroblasts (MEFs) were practically indistinguishable from their wild type counterparts, despite having defective nuclear morphologies, and identical ∆9*Lmna* expression levels to MAFs [45].

These findings revealed an unexpected difference between embryonic and post-natal fibroblasts in their susceptibility to ∆9*Lmna*, suggesting a possible explanation as to why progeric children are overtly normal at birth, since A-type lamins (and probably Progerin) are expressed during development [46-48]. One significant molecular difference between MEFs and MAFs is that MAFs express higher levels of the Wnt regulated transcription factor Lef1, compared to MEFs. Similar differences in LEF1 levels were also noted in normal human fibroblasts with 12-16 week old fetal fibroblasts expressing reduced levels of LEF1 compared to those from a 17-year old individual (unpublished observations). In the ∆9*Lmna* MAFs Lef1 levels were markedly reduced, due to ∆9*Lmna* inhibition of canonical Wnt signaling. LEF1 levels were also significantly reduced in fibroblasts established from two HGPS children, indicating that different truncated, farnesylated *LMNA* mutant proteins inhibit LEF1 expression and function. Some of the ECM genes, whose expression was reduced by ∆9*Lmna,* are transcriptionally regulated by Lef1 indicating a direct link between the inhibition of ECM gene expression and ∆9*Lmna*. The finding that ECM expression was significantly reduced in the ∆9*Lmna* model was similar to microarray studies on HGPS fibroblasts and MSCs, where ECM gene expression was also profoundly altered [48-50].

Loss of the endoprotease ZMPSTE24 results in the persistence of unprocessed, farnesylated, full length pre-laminA. Mice lacking ZMPSTE24 develop progeroid features which include skeletal abnormalities, alopecia and death by 6 months, [24,41,43]. In contrast to mice, the few humans identified with ZMPSTE24 deficiency, develop restrictive dermopathy (RD) or tight skin, resulting in perinatal mortality [51]. Cells, including MEFs, from *Zmpste24* null mice show increased cellular DNA damage, together with defective DNA damage responses, as well as defective Wnt signaling in the hair follicles [52]. The accelerated aging phenotype of *Zmpste24^−/−^* mouse was partly alleviated when crossed into a p53-deficient background [40] and completely rescued when made heterozygous for *Lmna* expression revealing a dosage effect of pre-laminA on the pathology [40]. Analysis of fibroblasts from one patient with RD also revealed persistent activation of DNA damage checkpoints [16]. Why ZMPSTE24 loss in humans results in a more severe (peri-natal) pathology than in mice is unclear, although it is possible that mice may be more “resistant” to *Zmpste24* deficiency because of their longer telomeres.These results reflect the *in vitro* results reported by Kudlow *et al*., and others [23,28] inwhich inhibition of p53 signaling rescued the Progerin-induced DNA damage and impaired proliferation. However, it is still unclear whether *Zmpste24*-deficiency damages telomeres in a similar way to Progerin.

In other progeroid mouse lines, *Lmna* was replaced with a variant allele that only expresses Progerin (*Lmna^HG/+^)*. At birth these mice were overtly normal with the *Lmna^HG/+^* heterozygotes showing retarded postnatal growth, weight loss, diminished adipose tissue, skeletal defects and death by nine months [53]. The few homozygotes produced, showed severe growth retardation and all died by three weeks. In contrast to *Zmpste24^−/−^* mice, skeletal muscle function was unaffected and, as with the ∆9*Lmna* mice, no pathology was detected in the ascending aorta, although the state of the great vessels was not reported. It is noteworthy that all three mouse lines show similar pathologies in mesenchymal tissues, with the skeletal system, being predominantly affected.

In addition to these three lines where the indigenous *Lmna* gene was modified, transgenic lines were derived in which Progerin was expressed as a transgene. In two of the transgenic lines Progerin was specifically expressed in the skin and resulted in abnormal nuclear morphologies and in one line, epidermal hyperplasia, hyperparakeratosis, hyperplasia of the sebaceous glands, culminating subcutaneous fat loss, dermal fibrosis and hypoplastic sebaceous glands and dental defects [54,55]. In the third line, no overt effect was noted, however, by one year, loss of vascular smooth muscle was detected in the aorta [42].

How do these findings explain that HGPS patients die in their early to mid teens from atherosclerosis and does this relate to normal aging? Post-mortem analyses of a few HGPS patients revealed extensive loss of vascular smooth muscle (VSM), particularly in the aorta and great vessels proximal to the heart and, in two others, extensive atherosclerosis [56,57]. In two progeroid mouse models, extensive loss of VSM in both the aorta and great vessels proximal to the heart was present. In the ∆9*Lmna* mutant mice, a rapid onset of VSM loss, together with increased apoptosis in the great vessels proximal to the heart was apparent at 2 weeks of age. These mice also exhibited extensive reduction in many ECM genes expressed in the skeletal system together with reduced trabecularity and minerality in the calvarial and axial skeleton, features consistent with the skeletal pathology found in HGPS. In the transgenic model [42], the human Progerin transgene resulted in loss of VSM in the aorta, but only after one year. Similarly, Werner's patients show accelerated atherosclerosis and death from myocardial infarction, despite normal circulating cholesterol levels. This suggests that cardiovascular defects are frequently associated with increased DNA damage and/or that the cardiovascular system is particularly sensitive or susceptible to DNA damage [58].

The ∆9*Lmna* mutant phenocopies many of the tissue, cellular and molecular pathologies characteristic of Progeria, yet, a number of interesting discrepancies exist. How does the ∆9*Lmna* mutation lead to decreased Wnt-signaling, and does this, in turn, exclusively reduce ECM synthesis? Progerin inhibits Wnt signaling to a lesser extent than the ∆9*Lmna* mutant, although progeric fibroblasts show reduced levels of Lef1 and treatment with a GSK-3 inhibitor improves their proliferation [45]. Moreover, what is the role of DNA damage in the ∆9*Lmna* mice since there was no evidence of increased genomic instability or an increase in H2A-X foci? If so, how, and to what extent could wild type ECM restore this phenotype? Are the farnesylated, truncated variants of LaminA, defective in Wnt signaling, telomere damage and disrupted ECM expression somehow interlinked? Some (or all?) of these parameters are clearly affected during normal ageing, but why are they especially critical to the vascular system? One common link maybe through the Forkhead box O (FoxO) transcription factor network. Oxidative stress activates these factors, which in turn inhibit Wnt mediated transcription by competing for β-catenin. Inhibition of FOXO mediated transcription factor activity improves VSM viability and promotes osteoblast differentiation [59].

Differences in telomere length and maintenance between mice and humans are an important consideration when generating mouse models for Werner syndrome and Ataxia telangectasia; in both cases Wrn-/- and Atm-/- deficient mice exhibited progeriod features only after they were crossed into a telomerase-deficient background [38,60]. However the role of DNA damage in the molecular pathology of HGPS remains open. Mouse models that seemingly do not show DNA damage still develop a progeroid phenotype. Furthermore, other progeroid/atypical Werner's cases, caused by *LMNA* mutations, have not been investigated as to whether these mutations result in DNA damage.

HGPS is complex, although some understanding of the molecular pathology has been gained by the study of patient-derived fibroblasts, ectopically expressed proteins in normal human fibroblasts and mouse models. Nevertheless, we still lack a clear picture how (and if) Progerin, Wnt signaling, the ECM, DNA damage and telomeres are interconnected. Two recently generated *in vitro* disease models for Progeria using induced pluripotent stem cells (iPSC) provide further insights [48,61]. iPSC do not express Lamin A (or Progerin), are overtly normal, and can differentiate into relevant tissues that are particularly affected in Progeria patients (i.e. mesenchymal lineages, vascular smooth muscle etc), and which cannot be derived from patients. From such “disease in a dish” studies it was apparent that VSM and mesenchymal stem cells were especially sensitive to Progerin, possibly because they expressed the highest levels of Progerin-LaminA, relative to other cell types, such as endothelial and neuronal lineages. It was noted that the HGPS-MSCs showed elevated levels of DNA damage and were far less efficient than normal MSCs at rescuing vascular circulation following ischemia [46]. In addition, comparison of gene expression profiles between MSCs derived from normal individuals and HGPS-MSCs, it was striking that Gene Ontology analysis revealed both Extracellular Matrix and Wnt signaling to be the most significant differences between the HGPS- and normal MSCs [48].

However the recurring question is do progeric diseases tell us something about the normal ageing process? In a recent study, VSM isolated from arteries from a limited number of 70-80 year old individuals, expressed elevated levels of pre-laminA, the farnesylated precursor of mature LaminA, whereas in the arteries from teenagers no pre-lamin A was detected [62]. This increase of pre-laminA was due to decreased levels of ZMPSTE24 in the VSM, possibly as a consequence of ZMPSTE24 expression being sensitive to oxidative stress. VSM cells appear to be particularly vulnerable to the anti-proliferative effects of pre-laminA, as suggested by VSM derived from HGPS iPS cells [48]. Why VSM should be seemingly sensitive to Progerin is unclear, but it maybe relevant that in skin biopsies from HGPS patients, VSM expressed the highest levels of Progerin [18,48]. Several studies have also suggested that low levels of Progerin protein may be detected in tissues from aged individuals [62,63]. It is conceivable that part of the normal aging process, is a decline in vascular integrity due to defective LaminA processing. Defective processing may arise either due to a decrease in ZMPSTE24 activity and/or defective splicing resulting in low but significant levels of Progerin being produced [63]. In addition, it remains to be established to what extent the other tissue pathologies in HGPS, arise either through autonomous cell expression of Progerin or are a consequence of the vascular defects. These issues and whether defective LaminA processing enhances telomeric DNA damage, inhibits Wnt signaling and ECM regulation, all of which may contribute to the normal process of aging, are all testable propositions.
